# Influence of sociodemographic factors on expert-rated non-technical skills in trauma team simulations

**DOI:** 10.1007/s00068-025-03076-2

**Published:** 2026-02-02

**Authors:** Marika Ylönen, Mikko Heinänen, Antti Tuominen, Juha Paloneva, Eerika Rosqvist

**Affiliations:** 1 Department of Anaesthesia and Intensive Care, Wellbeing Services County of Central Finland, Hospital Nova of Central Finland, Jyväskylä, Finland; 2 Institute of Clinical Medicine, Anaesthesiology and Intensive Care, University of Eastern Finland, Kuopio, Finland; 3 Department of Orthopaedics and Traumatology, Trauma Unit and Helsinki Trauma Registry, University of Helsinki and Helsinki University Hospital, Helsinki, Finland; 4 Department of Surgery, Wellbeing Services County of Central Finland, Hospital Nova of Central Finland, Jyväskylä, Finland; 5 University of Eastern Finland, Kuopio, Finland; 6 Oulu University Hospital and University of Oulu, Oulu, Finland; 7 Center of Healthcare Expertise, Competence and Development Services/Human Resources Services, Wellbeing Services County of Central Finland, Hospital Nova of Central Finland, Jyväskylä, Finland

**Keywords:** T-NOTECHS, Trauma team, Simulation training, Non-technical skills, Sociodemographic

## Abstract

**Purpose:**

Hospital trauma teams consist of multidisciplinary healthcare professionals with diverse backgrounds and varying levels of non-technical skills. While these skills can be improved through team simulation training, little is known whether there is a link between the sociodemographic backgrounds of team members and their non-technical skills. The objective of this study was to assess whether such sociodemographic details influence variations in non-technical skill levels as evaluated with the T-NOTECHS scale.

**Methods:**

This prospective study included 337 trauma team simulation trainings with 1822 participants in 2013-22. Data collection metrics included: (1) individual sociodemographic details (gender, age, occupation, working experience in years in present duty, and number of times participating in trauma team simulation training); (2) team size; and (3) team performance as assessed by an expert rater across the five domains of T-NOTECHS (leadership, cooperation and resource management, communication and interaction, assessment and decision making, and situation awareness/coping with stress) on a five-point Likert scale.

**Results:**

There were no significant differences in the T-NOTECHS scores based on gender, age, occupation, or training frequency. Work experience and team size were consistently associated with non-technical performance, with lower scores among less experienced professionals and smaller teams.

**Conclusion:**

Work experience and team size were the strongest background factors associated with non-technical skills in simulated trauma teams. Less experienced professionals and those in smaller teams had consistently lower performance. These findings underscore the importance of clinical experience and team composition in team effectiveness and highlight the need for targeted support for early-career professionals.

## Introduction

The in-hospital trauma team consists of a multidisciplinary group of individuals, who each provide simultaneous input into the assessment and management of the trauma patient. The primary aims of the team are to rapidly resuscitate and stabilize the patient, prioritize and determine the nature and extent of injuries, and prepare the patient for transport to the site of definitive care [1], ultimately reducing the time from the injury to critical interventions and surgery [1–3].

Errors in adult trauma resuscitation occur and are diverse. They can occur not only in technical procedures, but also in the team’s non-technical skills, such as communication [4–6]. Poor non-technical skills can lead to poorer medical care and can compromise patient safety [1, 7, 8]. An important way to reduce adverse events related to non-technical skills is through simulation training [2, 8, 9].

Objective assessment of learning outcomes and skill development of trauma teams in simulation trainings is essential [10], as these trainings are costly to conduct [11–13]. The modified NOn-TECHnical Skills scale for trauma (T-NOTECHS) is designed to evaluate teamwork-related behavioral aspects. It serves as a tool for teaching, assessing learning outcomes, and evaluating the non-technical skills of multidisciplinary trauma resuscitation teams [14, 15], demonstrating excellent reliability in overall scores [16]. T-NOTECHS has been used to show the impact of trauma team training on nontechnical skills both in real-life [7, 17, 18] and simulated trauma resuscitations [7, 19, 20]. However, it remains unclear whether differences in sociodemographic backgrounds of trauma team members influence their non-technical skill levels as assessed by T-NOTECHS.

Non-technical skills play an important role in improving the efficiency of trauma teams. We hypothesized that sociodemographic details of individuals influence the variations in non-technical skill levels in trauma team simulation training, as assessed by an expert rater using the T-NOTECHS scale.

## Methods

### Setting and design

The study was conducted in the level II/III trauma center Central Finland Hospital Nova Jyväskylä, Finland. Hospital Nova is an academic teaching hospital and has a 24-hour emergency department and is the only hospital in its catchment area (population of 273 000) that provides public secondary care. The real-life trauma team is activated in case of a (potentially) severely injured patient, which is predefined by physiological or anatomical criteria or mechanism of trauma. At our institution, the trauma team consists of a trauma team leader (a surgical resident or consultant), a surgical resident, an anesthetist, a radiologist, two emergency department nurses, and one rotation nurse. There are no differences in trauma team composition during the night or the day.

We started to conduct multidisciplinary trauma team simulation training in 2003 and regularly in 2009. In Hospital Nova, trauma team simulation training is for residents, consultants, and nurses working in the emergency department, intensive care unit, and operating room, and they act in their real-life professional roles in an emergency department room. Due to resource limitations, it was not always possible to gather the full seven-member trauma team for training, and in some cases, simulations were conducted with teams of four participants. The basics of our ad hoc and in situ trauma team simulation trainings have been described previously [12, 13, 19, 21]. The 2-hour multidisciplinary simulations started with an approximately 30-minute structured introductory talk on the aims of the training and the background information on the practiced topic. Although every simulation scenario had its own learning objectives, all simulations had a common goal of rehearsing and improving communication, leadership, and teamwork. The introductory talk was followed by familiarization with the patient simulator and the training environment. All simulations ended with a guided reflective learning conversation.

The main simulation instructor, who acted also as the expert rater (four different persons over the years) was an senior anesthetist and intensivist. This instructor was paired with nurse teachers, who acted as the simulator pilots. All instructors and nurse teachers completed a simulation educator training course and had long experience in clinical trauma, critical care, and simulation-based training. The design, conduct, recording, and reporting of simulations were performed in collaboration with the Center of Healthcare Expertise’s educational designers and technical experts.

We started data collection in 2013. Institutional review board approved the study. The data were collected prospectively and anonymously between October 2013 and December 2022. No real-life resuscitations were used for data collection in this study.

### Instruments

Sociodemographic data were collected before the trainings from the self-assessment questionnaire of 1822 participants (Table 1). We used a self-assessment questionnaire that has been published previously [19]. The questionnaire included the following five background questions: gender, age, occupation, working experience in years in the present duty, and number of times participating in a trauma team simulation training. The size of the teams was recorded. The self-assessment questionnaire was anonymous and optional. Teamwork was independently evaluated by the main instructor (expert rater) immediately after each simulation scenario using a modified NOTECHS scale for trauma (T-NOTECHS). The instructors (four different individuals over the years) were senior anaesthetists and intensivists. All had completed at least one simulation educator training course and had extensive clinical and teaching experience. The 5-point Likert-type scale comprises the following categories of teamwork behavior: leadership, cooperation and resource management, communication and interaction, assessment and decision making, and situation awareness/coping with stress. Score 1 represents poor team performance, and 5 represents ideal team behavior within the category. Thus, the total score ranges from 5 to 25 [14]. The translated, Finnish version has demonstrated fair reliability and good construct validity for assessing team performance in simulated multi-professional trauma team resuscitation [21], and this version has been used in our trauma team simulation trainings [12, 13, 19]. Overall team performance was evaluated by total T-NOTECHS score, the sum of all five category scores (scale 5–25). T-NOTECHS were introduced to expert raters, and concepts of behavior rating and specific features of this tool were discussed before commencing assessment. The expert rater evaluated a total of 337 in-situ simulated resuscitations. The ratings were not discussed with the participants. The raters were not blinded.

**Table 1:** Participant sociodemographic details (*N* = 1822)

	Frequency (%)
Gender	1807 (100)
Female	1067 (59)
Male	740 (41)
Age, years	1804 (100)
20–29	473 (26)
30–39	671 (37)
40–49	407 (23)
> 49	253 (14)
Occupation	1802 (100)
Physician	880 (49)
Nurse	992 (51)
Length of experience in present job, years	1775 (100)
0–2	674 (38)
3–5	237 (13)
> 5	864 (49)
Training frequency in simulations, times	1753 (100)
1	570 (32)
2–5	700 (40)
> 5	483 (28)
Team size	1822 (100)
≤ 4	353 (19)
> 4	1469 (81)

**Table 2:** A associations between sociodemographic characteristics and T-NOTECHS scores in 337 simulated trauma teams with 1822 participants (scale 1–5)

Group	Leadership			Cooperation and resource management			Communication and interaction		
	Mean (SD)	Mean difference (95% CI)	*p*-value	Mean (SD)	Mean difference (95% CI)	*p*-value	Mean (SD)	Mean difference (95% CI)	*p*-value
Gender									
Male	3.95 (0.801)	0.001 (-0.083, 0.085)	0.988	3.90 (0.634)	-0.036 (0.103, 0.030)	0.281	3.67 (0.626)	-0.006 (-0.072, 0.060)	0.086
Female (ref.)	3.95 (0.806)			3.91 (0.629)			3.68 (0.636)		
Age band, years									
20–29	3.94 (0.814)	-0.065 (-0.227, 0.097)	0.434	3.88 (0.628)	-0.050 (-0.178, 0.078)	0.446	3.66 (0.615)	-0.079 (-0.207, 0.048)	0.223
30–39	3.94 (0.796)	-0.021 (-0.157, 0.114)	0.759	3.91 (0.619)	-0.022 (-0.129, 0.085)	0.692	3.65 (0.621)	-0.023 (-0.130, 0.084)	0.672
40–49	3.98 (0.823)	-0.044 (-0.177, 0.090)	0.521	3.89 (0.661)	0.055 (-0.050, 0.161)	0.306	3.70 (0.663)	-0.024 (-0.129, 0.081)	0.651
≥50 (ref.)	3.97 (0.790)			3.96 (0.631)			3.70 (0.617)		
Occupation									
Physician	3.95 (0.799)	0.017 (-0.072, 0.106)	0.708	3.88 (0.626)	0.060 (-0.010, 0.130)	0.091	3.68 (0.634)	-0.003 (-0.073, 0.067)	0.935
Nurse (ref.)	3.96 (0.811)			3.94 (0.634)			3.67 (0.624)		
Work experience									
0–2	3.90 (0.766)	0.133 (0.007, 0.260)	**0.038**	3.86 (0.614)	0.133 (0.034, 0.233)	**0.009**	3.63 (0.615)	0.119 (0.020, 0.218)	**0.019**
3–5	3.96 (0.862)	0.076 (-0.056, 0.209)	0.257	3.88 (0.674)	0.105 (0.000, 0.209)	**0.049**	3.67 (0.640)	0.097 (-0.007, 0.201)	0.068
>5 (ref.)	3.99 (0.812)			3.96 (0.633)			3.72 (0.635)		
Training frequency									
1	3.91 (0.798)	0.024 (-0.096, 0.144)	0.699	3.87 (0.654)	0.021 (-0.074, 0.116)	0.663	3.61 (0.622)	0.040 (-0.055, 0.134)	0.410
2–5	3.97 (0.804)	-0.027 (-0.130, 0.076)	0.611	3.92 (0.628)	-0.019 (-0.101, 0.062)	0.642	3.69 (0.628)	-0.018 (-0.099, 0.063)	0.663
>5 (ref.)	3.99 (0.768)			3.93 (0.606)			3.70 (0.625)		
Team size									
≤4	3.91 (0.826)	0.057 (-0.040, 0.1549)	0.251	3.78 (0.620)	0.157 (0.080, 0.233)	**< 0.001**	3.63 (0.611)	0.054 (-0.022, 0.131)	0.166
>4 (ref.)	3.96 (0.798)			3.93 (0.631)			3.68 (0.633)		

The mean difference is significant at the 0.05 level. Reference categories: gender female, age ≥ 50 years, occupation nurse, work experience > 5 years, training frequency > 5 times and team size > 4 members

**Table 3:** Associations between sociodemographic characteristics and the T-NOTECHS scores in 337 simulated trauma teams with 1822 participants (scale 1-5, total scores 5-25)

Group	Assessment and decision making			Situation awareness/ Coping with stress			Total		
	Mean (SD)	Mean difference (95% CI)	*p*-value	Mean (SD)	Mean difference (95% CI)	*p*-value	Mean (SD)	Mean difference (95% CI)	*p*-value
Gender									
Male	3.84 (0.716)	-0.006 (-0.081, 0.069)	0.874	4.05 (0.698)	0.002 (-0.071, 0.076)	0.951	19.40 (2.797)	-0.045 (-0.340, 0.249)	0.762
Female (ref.)	3.86 (0.713)			4.04 (0.704)			19.43 (2.845)		
Age band, years									
20–29	3.87 (0.735)	-0.107 (-0.253, 0.038)	0.147	4.03 (0.702)	-0.088 (-0.230, 0.053)	0.222	19.38 (2.831)	-0.389 (-0.958, 0.179)	0.179
30–39	3.84 (0.701)	-0.054 (-0.176, 0.067)	0.381	4.05 (0.704)	-0.039 (-0.157, 0.080)	0.523	19.39 (2.764)	-0.159 (-0.635, 0.317)	0.513
40–49	3.85 (0.711)	-0.029 (-0.149, 0.091)	0.631	4.06 (0.695)	0.008 (-0.109, 0.125)	0.897	19.48 (2.919)	-0.035 (-0.504, 0.435)	0.885
≥50 (ref.)	3.85 (0.719)			4.08 (0.688)			19.57 (2.767)		
Occupation									
Physician	3.86 (0.713)	-0.001 (-0.080, 0.079)	0.986	4.04 (0.701)	0.026 (-0.052, 0.103)	0.518	19.41 (2.815)	0.065 (-0.246, 0.377)	0.681
Nurse (ref.)	3.85 (0.714)			4.07 (0.697)			19.48 (2.812)		
Work experience									
0–2	3.84 (0.717)	0.044 (0.070, 0.157)	0.450	3.99 (0.704)	0.153 (0.043, 0.263)	**0.007**	19.21 (2.749)	0.582 (0.140, 1.025)	**0.010**
3–5	3.86 (0.708)	0.008 (-0.111, 0.126)	0.900	4.06 (0.678)	0.086 (-0.030, 0.202)	0.145	19.44 (2.876)	0.371 (-0.092, 0.835)	0.116
>5 (ref.)	3.86 (0.713)			4.10 (0.700)			19.63 (2.832)		
Training frequency									
1	3.81 (0.714)	0.079 (-0.028, 0.187)	0.148	4.02 (0.719)	0.021 (-0.085, 0.126)	0.701	19.22 (2.822)	0.184 (-0.237, 0.606)	0.391
2–5	3.85 (0.727)	0.034 (-0.058, 0.127)	0.468	4.05 (0.691)	-0.018 (-0.108, 0.073)	0.704	19.49 (2.828)	-0.047 (-0.410, 0.315)	0.798
>5 (ref.)	3.87 (0.689)			4.07 (0.692)			19.56 (2.725)		
Team size									
≤4	3.78 (0.746)	0.078 (-0.009, 0.166)	0.079	3.92 (0.754)	0.170 (0.085, 0.255)	**< 0.001**	19.02 (2.963)	0.516 (0.175, 0.858)	**0.003**
>4 (ref.)	3.87 (0.706)			4.08 (0.683)			19.53 (2.770)		

Mean difference is significant at the 0.05 level

### Statistical analysis

Data were analyzed using IBM SPSS Statistics, version 29.0 (IBM Corp., Armonk, NY, USA). Demographic variables (age, gender, occupation, work experience, and training frequency) were described by frequency and percentage distributions. Differences in T-NOTECHS domains (leadership, cooperation and resource management, communication and interaction, assessment and decision making, and situation awareness/coping with stress) and total scores across participant characteristics were examined using a univariate linear model. Group-specific means and standard deviations were reported. Differences between groups were expressed as mean differences with corresponding 95% confidence intervals (CI) and calculated in reference to the designated comparison (reference) group. Gender, age group, occupation, work experience, training frequency, and team size were treated as categorical variables with the highest category used as the reference group for ordinal variables, and “nurse” and “female” used as reference categories for occupation and gender, respectively. *p*-values < 0.05 were considered statistically significant.

## Results

Participants’ sociodemographic details are presented in Table 1. There were no significant differences in T-NOTECHS scores based on gender, age, occupation, or training frequency (Tables 2 and 3). Work experience and team size were associated with significant differences in several domains. Participants with 0–2 years of work experience scored significantly lower points than those with > 5 years of experience in leadership (mean difference = 0.13, *p* = 0.038), cooperation and resource management (mean difference = 0.13, *p* = 0.009), communication and interaction (mean difference = 0.12, *p* = 0.019), situation awareness/coping with stress (mean difference = 0.15, *p* = 0.007), and in the total T-NOTECHS score (mean difference = 0.58, *p* = 0.010). Participants from smaller teams (≤ 4 members) scored significantly lower than those from larger teams in cooperation and resource management (mean difference = 0.16, *p* < 0.001), situation awareness/coping with stress (mean difference = 0.17, *p* < 0.001), and total score (mean difference = 0.52, *p* = 0.003) (Tables 2 and 3).

## Discussion

In this study, team size and work experience had the most consistent and statistically significant associations with non-technical skills assessed by an expert rater. Participants with < 2 years of work experience scored significantly lower in overall performance and in all non-technical skills, except for assessment and decision making. Similarly, members of smaller teams (≤ 4 people) consistently scored lower points in almost all measured non-technical skills and the total T-NOTECHS score. Other variables, such as gender, age, occupation, and training frequency, showed no statistically significant differences across domains.

Resuscitation teams consist of professionals from diverse backgrounds, and each member has specific roles [1, 22] and differing levels of expertise and knowledge [23]. Effective collaboration in emergency settings is essential [23], despite the challenges posed by briefly assembled ad hoc teams consisting of various specialties and occupations, which can affect interpersonal dynamics [9, 17, 24]. According to this study’s findings, trauma team members may compensate for individual weaknesses, enabling strong performance overall. This finding warrants further research.

Literature on the association between increasing age and non-technical skills is limited. In this study, age was not significantly associated with any of the assessed non-technical skill domains. However, slight numerical trends suggested slightly higher total scores among older participants, although these were not statistically significant. Jonsson et al. [25] found that greater age was significantly associated with better total team performance (β = 0.35, *p* = 0.04), teamwork (β = 0.04, *p* = 0.04), and task management (β = 0.04, *p* = 0.05), and a higher overall rating for global team performance (β = 0.09, *p* = 0.02) in a simulated ICU setting using the TEAM instrument and the ABCDE checklist. Additionally, age was associated with team performance (β = 0.02, *p* = 0.04). Also, in our previous study, age was associated with self-assessed non-technical skills following in situ trauma team simulation [26]. These findings suggest that greater age may contribute to better non-technical skills when caring for critically ill patients in a simulated environment. Further research is needed to explore this relationship.

Gaining work experience is a key factor in professional development [26–28]. In this study, participants with limited work experience (< 2 years) had significantly lower total T-NOTECHS scores and lower scores across several domains, including leadership, cooperation and resource management, communication and interaction, and situation awareness/coping with stress, than those with > 5 years of experience. These results are consistent with the study of Pucher et al. [17], where they observed non-technical skills among 21 clinicians ranging from residents to senior attendees. They reported that T-NOTECHS scores improved with experience, with the junior cohort scoring significantly lower than the expert cohort (14.0 ± 2.0 vs. 21.5 ± 3.7, *p* = 0.003). This finding highlights the need to support early-career healthcare professionals through targeted training and mentoring to facilitate development of essential non-technical competencies. The role of experience is also a key factor for effective team performance in simulated healthcare settings. Integrating team-based skill development into workplace training, expanding simulation beyond trauma scenarios, and ensuring regular participation—supported by physician mentors and mentorship programs for nursing staff—can help develop essential non-technical competencies and enhance overall team performance.

Although previous studies have shown that regular training plays a crucial role in skill development [19, 29, 30] and that prior team training positively affects task performance [25], our study found no significant association between training frequency and non-technical skills. Participants may already have had high baseline levels of non-technical skills, leaving little room for measurable improvement regardless of training frequency. In this study, 27.6% of participants had participated in training more than five times, indicating a relatively high level of prior exposure, while only 32.5% were training for the first time. It is also possible that the tools we used to assess non-technical skills were insufficiently sensitive to detect small but meaningful improvements related to training frequency.

In this study, participants from smaller teams reported significantly lower scores than those from larger teams in cooperation and resource management, situation awareness/coping with stress, and total T-NOTECHS score. However, team size is an important factor to consider when designing team training and selecting assessment tools such as T-NOTECHS, as previous research suggests that evaluating non-technical skills may become less reliable in large teams (8–9 members) during trauma simulation training [31]. At our hospital, the trauma protocol assigns specific tasks to each participant. When training with a fully staffed team, individuals can concentrate on their designated roles, which may potentially lead to clearer and more efficient performance. Based on these observations, we now conduct simulation trainings only with fully staffed teams, as smaller groups limit role practice and task coverage. Training initially involved four-member teams, but standard team size has increased as trauma protocols evolved. These findings suggest the importance of aligning team composition with both training objectives and assessment methods to ensure meaningful evaluation of non-technical skills.

In this study, gender and occupation were not associated with differences in non-technical skills. We did not identify previous studies on the relationship between professional role, gender, and non-technical skill learning outcomes assessed by T-NOTECHS in team simulation training. This highlights an area of focus for future research, particularly using diverse study designs or assessment methods.

This study has limitations. First, we used real-time assessments rather than video recordings, which may lower reliability [15], as T-NOTECHS is more reliable in video reviews [16, 32]. Video recording was not feasible due to practical constraints, including limited training time, absence of suitable technical equipment, and instructors’ lack of training in video handling. Moreover, obtaining trainee consent for recordings could have reduced participation. Consequently, instructor effort was prioritized for real-time assessment. Further evidence is needed to validate the scale’s use in real-time evaluations [15].

Second, the T-NOTECHS scale assesses the multidisciplinary trauma team, not individual performances [7]. To address this, we have collected both self-assessment questionnaires on individual backgrounds and T-NOTECHS ratings since 2013, allowing us to combine unique datasets and explore potential links between individual characteristics and team-level non-technical skills. However, due to the lack of prior research, our main aim was exploratory: to examine whether associations exist between individual sociodemographic factors and levels of non-technical skills.

Another limitation is that only one rater’s evaluation was used per simulation training, which may introduce single-source bias. Our earlier study reported poor-to-fair interrater reliability (ICC 0.30–0.41) [19], consistent with the findings of Steinemann et al. [14] (ICC 0.44) in real-time simulation assessments. Using at least two raters might reduce bias and provide consensus scoring [21, 33]. However, multiple raters may focus on different behaviors, potentially affecting the results. In practice, using a single rater is considered acceptable for quality assessments, as the T-NOTECHS overall score is considered reliable [16]. Due to resource constraints, only one expert rater was used per simulation, although over the nine-year study period, four different instructors served as raters, potentially providing some variation in assessments. Given that raters applied standardized T-NOTECHS criteria and did not share their assessments with participants, the absence of blinding is unlikely to have introduced systematic bias. As the data were primarily collected to improve trauma simulations, raters were not aware at the time of training that their evaluations would later be used for research. Although, informed consent for the use of their assessments was obtained thereafter, further ensuring transparency and ethical conduct.

Additionally, a limitation of this study is the use of only univariate statistical models. This approach was selected due to the exploratory aim and modest sample size, which made multivariable modelling prone to overfitting. However, without multivariable adjustment, potential confounding cannot be fully accounted for, and the findings should be interpreted cautiously.

A strength within this limitation is that we had four different raters in this study, which reduces systemic bias by incorporating diverse perspectives and large datasets. Moreover, similar tools like the Oxford NOTECHS scale have demonstrated that reliable team evaluations can be conducted by a single trained observer from various professional backgrounds [34]. Finally, most of our simulations and assessments were conducted before recent reviews on non-technical skill assessment tools [8, 15, 35].

The strengths of this study include the large dataset of 1822 participants from both novice and expert healthcare professionals and a 9-year study period. Another key strength is the development of a Finnish version of the T-NOTECHS scale [21], which is routinely used in our trauma team simulation trainings [12, 13, 19]. We also used expert raters, which improves the reliability of the assessments [15]. Other strengths include the thorough training of raters to evaluate team performance, the real hospital environment in which the assessments were conducted, and the variety of simulations assessed, which improves generalizability of the results.

## Conclusions

Work experience and team size were the most influential background factors associated with better non-technical skills assessed by expert raters. Less experienced professionals and those working in smaller teams consistently scored lower in non-technical performance. These findings highlight the critical role of both clinical experience and team structure in formulating effective team behavior in simulated trauma teams. Other variables, including gender, age, occupation, and training frequency, did not show associations with T-NOTECHS scores. Further research is needed to explore how to best support early-career professionals in acquiring crucial team competencies.

## Data Availability

The dataset supporting the conclusions of this article can be obtained upon reasonable request from the corresponding author.
